# Nylon-Based Composite Gel Membrane Fabricated via Sequential Layer-By-Layer Electrospinning for Rechargeable Lithium Batteries with High Performance

**DOI:** 10.3390/polym12071572

**Published:** 2020-07-15

**Authors:** Sainan Qin, Yuqi Wang, Xu Wu, Xingpeng Zhang, Yusong Zhu, Nengfei Yu, Yi Zhang, Yuping Wu

**Affiliations:** School of Energy Science and Engineering, Nanjing Tech University, Nanjing 211816, China; qinsn@njtech.edu.cn (S.Q.); yuqiwang@njtech.edu.cn (Y.W.); 3503150120@njtech.edu.cn (X.W.); 3503150127@njtech.edu.cn (X.Z.); yunf@njtech.edu.cn (N.Y.)

**Keywords:** electrospinning, gel polymer electrolytes (GPEs), lithium ion batteries (LIBs), nylon, sandwich-structured composite

## Abstract

With the raw materials of poly(vinylidene-co-hexafluoropropylene) (P(VDF-HFP)) and polyamide 6 (PA6, nylon 6), a sandwich-structured composite membrane, PA6/P(VDF-HFP)/PA6, is fabricated via sequential layer-by-layer electrospinning. The nylon-based composite exhibits high absorption to organic liquid electrolyte (270 wt%) owing to its high porosity (90.35%), good mechanical property (17.11 MPa), and outstanding shut-down behavior from approximately 145 to 230 °C. Moreover, the dimensional shrink of a wet PA6 porous membrane immersed into liquid electrolyte is cured due to the existence of the P(VDF-HFP) middle layer. After swelling by the LiPF_6_-based organic liquid electrolyte, the obtained PA6/P(VDF-HFP)/PA6-based gel polymer electrolytes (GPE) shows high ionic conductivity at room temperature (4.2 mS cm^−1^), a wide electrochemical stable window (4.8 V), and low activation energy for Li^+^ ion conduction (4.68 kJ mol^−1^). Benefiting from the precise porosity structure made of the interlaced electrospinning nanofibers and the superior physicochemical properties of the nylon-based composite GPE, the reversible Li^+^ ion dissolution/deposition behaviors between the GPE and Li anode are successfully realized with the Li/Li symmetrical cells (current density: 1.0 mA cm^−2^; areal capacity: 1.0 mAh cm^−2^) proceeding over 400 h at a polarization voltage of no more than 70 mV. Furthermore, the nylon-based composite GPE in assembled Li/LiFePO_4_ cells displays good electrochemical stability, high discharge capacity, good cycle durability, and high rate capability. This research provides a new strategy to fabricate gel polymer electrolytes via the electrospinning technique for rechargeable lithium batteries with good electrochemical performance, high security, and low cost.

## 1. Introduction

With the rapid development of portable electronic devices and electric vehicles (EVs), lithium ion batteries (LIBs) are considered as one of the attractive power sources owing to their high output voltage, high energy density, good cycling performance, and environmental friendliness [1]. However, LIBs still can not satisfy the increasing application requirements because of the following shortages: (1) The wide application of carbonate-based organic liquid electrolyte leads to the safety issues of battery systems [2]. (2) The limited theoretical capacity (372 mAh g^−1^) of the conventional graphite-based anode in LIBs can not satisfy the increasing expectations of high specific capacity [3]. From developing new manufacture processes to seeking novel electrolyte and electrode materials, various efforts have been devoted to enhancing the security and energy density of LIBs [4]. Gel polymer electrolytes (GPEs) incorporating the organic liquid electrolyte in the polymer matrix are regarded as one of the feasible strategies to improve the safety and the specific capacity of LIBs due to their outstanding characteristics of high ionic conductivity at ambient temperature, wide electrochemical window, good interfacial compatibility with the electrodes, excellent thermal stability, and probable Li dendrite suppression [5]. The specific capacity of LIBs can be improved greatly by using Li (lithium) metal anode due to its unrivaled theoretical capacity of 3860 mA h g^−1^, the lowest redox voltage (−3.04 V versus SHE (standard hydrogen electrode)), and a smaller density (0.534 g cm^−3^) [6]. Nevertheless, the uncontrolled growth of metal dendrites during the repeated plating/stripping processes of the Li^+^ ions on the surface of Li metal will result in safety concerns and a gradual degradation of cycling efficiency, which hinders the practical usage of Li anodes [7].

Among the polymer hosts of GPEs researched, poly(vinylidene-co-hexafluoropropylene) (P(VDF-HFP)) has attracted the wide interests of the scientific community due to its appealing properties such as high dielectric constant (ε ≈ 9.4–10.6), good electrochemical stability, excellent electric insulation, superior flame retardation, and easy film formation [8]. However, some drawbacks such as the insufficient mechanical strength after swelling by organic liquid electrolytes, high cost, and some unpredictable side reactions between P(VDF-HFP) and Li metal at high temperature restrain the wide application of the kind of GPE in LIBs [9]. Polyamide 6 (PA6), also called nylon 6, is widely known as an excellent engineering thermoplastic polymer for its superior chemical resistance, high tensile strength, good elasticity, high thermal stability, spinnability, and low cost. In addition, PA6 membranes always show high uptake to organic liquid electrolytes for abundant amide groups in the backbone of the macromolecule [10]. However, the severe dimension shrinkage of wet nylon-based separators (the nylon-based separators saturated by the carbonate-based organic liquid electrolytes) holds back their wide application in energy storage devices [11].

Amounts of electrospun materials have been reported to be applied as anodes, cathodes, or separators for secondary batteries [12]. Electrospinning is a diffusely applied and versatile technique for fabricating functional porous fibrous membranes with controllable compositions and structures. The obtained electrospun polymer membranes with high porosity can accommodate large quantities of liquid electrolytes and offer effective conduction channels, which are beneficial to high ionic conductivity and good electrochemical performance [13,14].

In this work, a sandwich-structured nylon-based composite GPE (denoted as PA6/P(VDF-HFP)/PA6) is fabricated with a sequential layer-by-layer electrospinning technique to realize uniform Li^+^ ion electrodeposition and suppress the formation of lithium dendrites. The electrospun nylon-based composite membrane exhibits high absorption to organic liquid electrolyte, good mechanical property, and outstanding shut-down behavior. Owing to the existence of a P(VDF-HFP) layer in the composite, the dimension shrinkage of the wet PA6 membrane can be successfully cured. The PA6/P(VDF-HFP)/PA6-based GPE swollen by the LiPF_6_-based organic liquid electrolyte shows high ionic conductivity, a wide electrochemical window, and low activation energy for ion conduction. The formation and growth of lithium dendrites in Li metal batteries can be successfully inhibited by the nylon-based GPE due to enabling to realize the homogenous Li^+^ ion dissolution/deposition between the electrolyte and the Li anode. Furthermore, the composite GPE in the assembled Li/LiFePO_4_ cells displays good electrochemical performance such as high discharge capacity, good cycle durability, and high rate capability. This research provides a new strategy to fabricate gel polymer electrolytes via the electrospinning technique for rechargeable lithium batteries with good electrochemical performance, high security, and low cost.

## 2. Experimental

### 2.1. Materials

PA6 and P(VDF-HFP) were purchased from Sigma-Aldrich (Shanghai, China) and dried in a vacuum at 80 °C for 24 h before use. A.R. (analytical reagent) grade formic acid (FA), acetic acid (Ac), N, N-dimethylacetamide (DMAc), and acetone were obtained from Aladdin (Shanghai, China) to dissolve the polymers. Al foil (approximately 20 mm), Li–metal foil, commercial separator of Celgard 2400 (polypropylene, PP), and LiFePO_4_ powder were acquired from Sinopharm (Shanghai, China) for cell assembling. LiPF_6_-based organic liquid electrolyte (the LiPF_6_ salt dissolved into EC (ethylene carbonate)/DMC (dimethyl carbonate)/EMC (ethyl methyl carbonate), 1/1/1 for *w*/*w*/*w*, 1 mol L^−1^) was supplied by Zhangjiagang Guotai-Huarong New Chemical Materials Co, Ltd. (Suzhou, China) All chemical materials were used as received without further purification.

### 2.2. Preparation of PA6/P(VDF-HFP)/PA6 Membrane

**Scheme 1:**
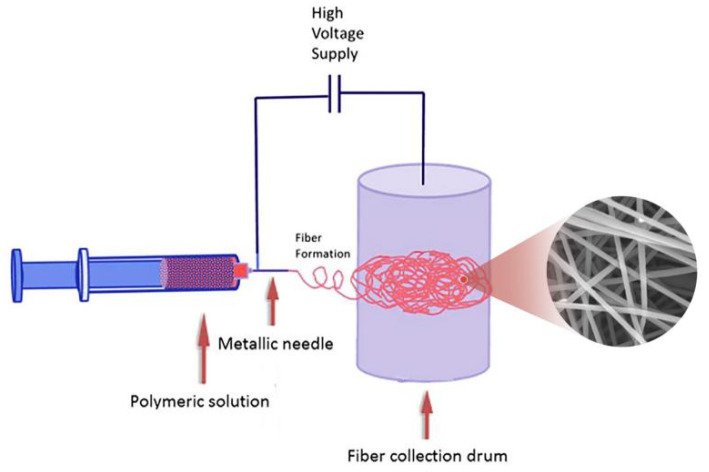
The schematic illustration of the preparation process for electrospun PA6/P(VDF-HFP)/PA6 membrane.

P(VDF-HFP) solution with a concentration of 15 wt % and PA6 solution with a concentration of 12 wt % were prepared by dissolving P(VDF-HFP) into DMAc and dissolving PA6 into FA/Ac (1/1, *v*/*v*) at room temperature (r.t.), respectively. Then, the PA6/P(VDF-HFP)/PA6 composite membrane was fabricated with the PA6 and P(VDF-HFP) solutions by the electrospinning equipment (Ucalery Elite-1), as shown in Scheme 1. The model of syringes, the inner diameters of the syringe needles, the translation distance of needles, the receiving distance, the operating temperature, and the relative humidity of the electrospinning process were fixed at 5 mL, 600 μm, ±3 cm, 15 cm, 25 °C, and 40%, respectively. The operating parameters of the electrospinning equipment were set as follows: 0.8 mL h^−1^ and 20 KV for the PA6 layer, and 1 mL h^−1^ and 15 KV for the P(VDF-HFP) layer. The electrospun PA6, P(VDF-HFP) and PA6 fibers were layer by layer collected on the same target rotating collector. The collecting time for each layer is 4 h. Then, the obtained free-standing PA6/P(VDF-HFP)/PA6 composite membranes were dried at 80 °C under vacuum for 12 h to remove residual solvents for further use.

### 2.3. Physical Characterization

The following measurements were performed at r.t unless otherwise stated.

The surface and the cross-section morphology of the electrospun membrane and commercial separator were observed by scanning electron microscopy (SEM, Phenom Prox, Eindhoven, The Netherlands) after being gold-sprayed. The samples were subjected to brittle fracture in liquid nitrogen for the cross-section observation. The mechanical properties of the materials were measured with the Sansi UTM4000 universal testing machine (SUNS, Shenzhen, China) based on GB/T 1040.3-2006 [15]. Differential scanning calorimetry (DSC) of the membranes were performed by a TG-DSC simultaneous thermal analysis system (NETZSCH-STA409, NETZSCH Group, Selb, Germany).

After the dry composite membranes or separator were immersed in *n*-butanol for more than 8 h, the porosity (P) was evaluated according to the following Equation (1):

(1) $$P=\frac{w_{2}-w_{1}}{\rho_{b}V_{m}}$$

where *w*_1_ and *w*_2_ represent the weights of the membranes or separator before and after saturation by n-butanol, respectively, ρ_b_ is the density of *n*-butanol (0.8098 g cm^−3^), and *V_m_* is the volume of the dry membrane, which is calculated with the radius and the thickness of the samples.

The uptake (*η*) to the LiPF_6_-based organic liquid electrolyte of the membranes can be calculated by the following Equation (2):

(2) $$\eta =\frac{w_{t}-w_{0}}{w_{0}}\times 100\%$$

where *w*_0_ is the weight of the dry membrane, and *w_t_* is the weight of the wet membrane, which was absorbed the LiPF_6_-based organic liquid electrolyte for more than 8 h.

### 2.4. Electrochemical Measurement

The following measurements were performed with an electrochemical work station of Chenhua CHI660C (Chenhua, Shanghai, China) except for the characterization of the electrochemical performance of Li/LiFPO_4_ cells.

Using the blocking-type cells with stainless steels (SSs) as electrodes, the electrochemical impedance spectroscopy (EIS) of the GPEs or wet separator at different temperatures (approximately 25–75 °C) was examined with the frequency range of 10–100 kHz (step potential: 10 mV). Based on the results of EIS, the ionic conductivity of the electrolytes was calculated according to Equation (3):

(3) $$\sigma =\frac{l}{R_{b}\cdot A}$$

where *l* represents the thickness of the membrane or the commercial separator, *R_b_* represents the bulk resistance from ESI, and *A* is the contact area between the membrane or the commercial separator and the SS electrode.

The lithium ion transference number (*t_Li+_*) of the electrolytes (the GPE or the organic liquid electrolyte) was obtained based on the Evans’ technique and calculated with Equation (4) [16].

(4) $$t_{{Li}^{+}}=\frac{I_{s}\left(\Delta V-I_{0}R_{0}\right)}{I_{0}\left(\Delta V-I_{s}R_{s}\right)}$$

The initial current (*I*_0_) and the steady state current (*I_s_*) were obtained from the chronoamperometry with a step potential (∆*V*) of 10 mV. *R*_0_ and *R_s_* represent the resistance of the cells (Li/GPE or wet separator/Li) before and after the chronoamperometry test. The resistance was acquired from the EIS of the Li/GPE or wet separator/Li blocking-type cells (frequency range: 10–100 kHz; step potential: 10 mV).

The electrochemical stability windows of the electrolytes were measured on the Li/GPE or wet separator/SS cells by linear sweep voltammetry (LSV) which was carried out at the potential range of 0–6.0 V (vs. Li^+^/Li) with a scan rate of 1 mV S^−1^.

A galvanostatic cycling test was performed with the symmetric cells (Li/wet separator or GPE/Li) to evaluate the Li^+^ ion plating/stripping stability between the Li/electrolyte interface. The symmetrical cells were cycled at a constant current density of 1.0 mA cm^−2^ and an areal capacity of 1.0 mAh cm^−2^.

The electrochemical performance of GPE was investigated with CR2025-type coin cells (Kejing, Hefei, China) on a Land battery test system. The coin cells were assembled by sandwiching GPE between the Li metal anode and the LiFePO_4_ cathode (LiFePO_4_/acetylene black/PVDF, 80/10/10, *w*/*w*/*w*) in an argon-filled glove box (water content < 1) ppm; oxygen content < 1 ppm). For researching the cycle behavior, the cells were charged and discharged at the current density of 1C (170 mA g^−1^) constantly between 2.5 and 4.2 V (vs. Li^+^/Li). For the rate evaluation, the cells were run for 5 cycles under the current densities of 0.2 C, 0.5 C, 1 C, 3 C, 5 C, and then back to 0.2 C.

## 3. Results and Discussion

**Figure 1:**
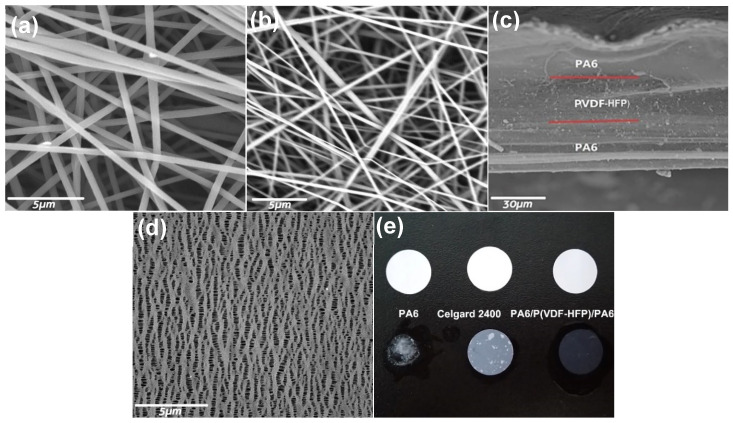
SEM images of (**a**) the surface of the P(VDF-HFP) electrospinning membrane, (**b**) the surface, and (**c**) the cross-section of the PA6/P(VDF-HFP)/PA6 electrospinning membrane as well as (**d**) the surface of a commercial Celgard 2400 separator. (**e**) A comparison photograph of dimension shrinkage of wet separator and electrospun membranes.

The morphology of the obtained electrospinning membranes and the commercial Celgard 2400 separator are presented in Figure 1. The interlaced network and nearly straightened fibers without any beads or particle aggregations are observed for all electrospinning membranes. The average diameters of the P(VDF-HFP) fibers are about 500 nm (Figure 1a), which are bigger than those of the PA6 fibers (approximately 200 nm, Figure 1b). It has been discovered that some factors such as the dielectric constant of the macromolecules, the concentration of the polymeric solutions, the distance between the nozzle tip and the collector, the operating voltage, and so on have great impact on the morphology and structure of the electrospinning fibers [17]. So, the distinctions of the morphology of P(VDF-HFP) and PA6 fibers can be ascribed to the differences of the physical properties, the concentration of the polymeric solutions, and the electrospinning voltage. From the cross-section of PA6/P(VDF-HFP)/PA6 membrane (Figure 1c), the composite seems very uniform via the sequential layer-by-layer electrospinning, and there is no obvious boundary between the PA6 layers and P(VDF-HFP) layer. The thickness of the three-layered membrane is about 50 μm (PA6 is approximately 15 μm and P(VDF-HFP) is approximately 20 μm), which is about double that of a Celgard 2400 separator [18]. From Figure 1d, the surface morphology of a commercial Celgard 2400 separator fabricated with the dry process is totally different from that of all the electrospun membranes. The commercial PP separator offers uniform pore structures with the size of 0.03–0.1 µm and the porosity calculated with Equation (1) is about 49.9% [19]. In addition, the dimension shrinkage of the wet membranes or separator is also evaluated, and the results are displayed in Figure 1e. The PA6 membrane huddles together after saturation by LiPF_6_-based liquid electrolyte. The PA6/P(VDF-HFP)/PA6 membrane and the commercial separator show the same dimensional stability after absorbing abundances of organic liquid electrolytes. From the research, the existence of a P(VDF-HFP) layer in the sandwich-structured composite is very crucial for keeping the dimensional stability of the PA6 layers.

**Figure 2:**
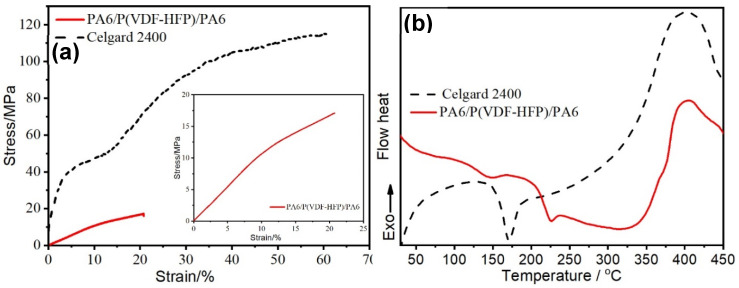
(**a**) The strain–stress curves of the PA6/P(VDF-HFP)/PA6 membrane and the Celgard 2400 separator. (**b**) Differential scanning calorimetry (DSC) curves of the PA6/P(VDF-HFP)/PA6 membrane and the Celgard 2400 at a temperature rising rate of 5 °C min^−1^ from 30 to 450 °C under air.

For satisfying the requirement of assembly and improving the security of batteries, the GPEs or separators want to own the appropriate tensile strength [20]. The mechanical properties of the PA6/P(VDF-HFP)/PA6 membrane and the Celgard 2400 separator are obtained by a tensile test, and the results are shown in Figure 2a. The tensile strength and the breaking elongation ratio of the electrospinning membrane are 17.1 MPa and 20.8%, respectively. Correspondingly, the tensile strength and the breaking elongation ratio of the Celgard 2400 separately are 115.3 MPa and 61.1%, which are consistent with the literature [21,22]. From the results, the mechanical property of the obtained composite membrane is poorer than that of the commercial PP separator. However, the tensile strength of the fabricated PA6/P(VDF-HFP)/PA6 membrane is greater than that of many of the reported separators/quasi-solid polymer electrolytes [23,24,25,26]. Therefore, the mechanical strength of the obtained PA6/P(VDF-HFP)/PA6 membrane is acceptable for application in LIB systems.

The thermal stability of the separator is directly related to the operating temperature, the electrochemical performance, and the safety of LIBs. The thermal stability of the PA6/P(VDF-HFP)/PA6 membrane and the Celgard 2400 separator was evaluated by differential scanning calorimetry (DSC) at a temperature rising rate of 5 °C min^−1^ from 30 to 450 °C under air. As displayed in Figure 2b, the phase-transition temperature of the Celgard 2400 separator is about 170 °C, which is the melting temperature of the raw material for the separator (ultra-high molecular weight polypropylene, UHMWPP) [27]. There are two phase-transition points at 145 °C and 230 °C in the DSC of the PA6/P(VDF-HFP)/PA6 membrane, corresponding to the melting temperature of P(VDF-HFP) and PA6 [10,28], respectively. That is, the electrospun PA6/P(VDF-HFP)/PA6 membrane will exhibit a shut-down behavior when the temperature is over 145 °C. However, the dimensional stability of the composite membrane still can be maintained by the PA6 layers to 230 °C. The shut-down behavior of the PA6/P(VDF-HFP)/PA6 membrane can greatly enhance the security of the battery systems, just as the commercial Celgard 2320 or 2325 separator (PP/PE/PP, shut-down temperature: 140-170 °C) can. In addition, all the samples including the electrospun membrane and the commercial separator will decompose when heated to 400 °C in the air (Figure 2b).

**Figure 3:**
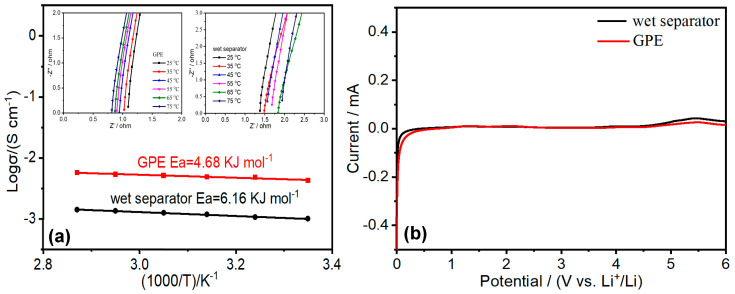
(**a**) Arrhenius plots of PA6/P(VDF-HFP)/PA6-based GPE and wet separator. Insets are the impedance plot of the conductivity data of the PA6/P(VDF-HFP)/PA6-based GPE and wet separator from 25 to 75 °C. (**b**) Linear sweep voltammograms of the PA6/P(VDF-HFP)/PA6-based GPE and wet separator at the scan rate of 1 mV s^−1^.

Electrospinning is always regarded as one of the efficient techniques to fabricate polymer membranes with high porosity. The higher porosity of the membranes will exhibit the higher uptake to organic liquid electrolytes. The porosity of the obtained nylon-based composite membrane (90.35%) is almost double of that of the PP separator (49.86%). Therefore, the uptake to LiFP_6_-based electrolyte of the electrospun nylon-based composite membrane can be up to 273 wt%, which is far more than that of Celgard 2400 (78 wt%). The absorbed abundances of LiPF_6_-based liquid electrolytes in the polymer matrix can support the migration and diffusion of Li^+^ ions with high concentration. As expected, the ionic conductivity of PA6/P(VDF-HFP)/PA6-based GPE at r.t. calculated with the EIS (Figure 3a) can reach up to 4.2 mS cm^−1^ [29], which is far beyond that of the PP separator saturated by LiPF_6_-based liquid electrolyte (1.0 mS cm^−1^). The good result mainly attributes to special three-layered structure and the high uptake to liquid electrolyte of the electrospun membrane.

The ionic conductivity of PA6/P(VDF-HFP)/PA6-based GPE and wet separator at different temperatures (25–75 °C) can be calculated using the EIS shown in the insets of Figure 3a. Based on the obtained results, the ionic conductivity of nylon-based composite GPE and wet separator increases with temperature. The dependence of ionic conductivity on temperature can be reasonably explained by Arrhenius ion conduction mechanism (*σ* = *A*exp(−*Ea/RT*)). The approximate linear relationship between Logσ and 1000/T is shown in Figure 3a. The obtained activation energy (*Ea*) is 4.68 kJ mol^−1^ for PA6/P(VDF-HFP)/PA6-based GPE and 6.16 kJ mol^−1^ for wet separator, respectively. The low activation energy of the composite GPE can be ascribed to its high uptake to liquid electrolyte. A great amount of organic liquid electrolytes in PA6 and P(VDF-HPF) matrix can quickly transfer the Li^+^ ions with high concentration.

The electrochemical window of PA6/P(VDF-HFP)/PA6-based GPE is about 4.8 V (Shown as Figure 3b), which can meet application requirements of the LIBs. The GPE and the wet separator exhibit the same electrochemical stability since both of them are saturated with LiFP_6_-based organic liquid electrolyte (LiFP_6_ dissolved into carbonate-based organic solvents).

**Figure 4:**
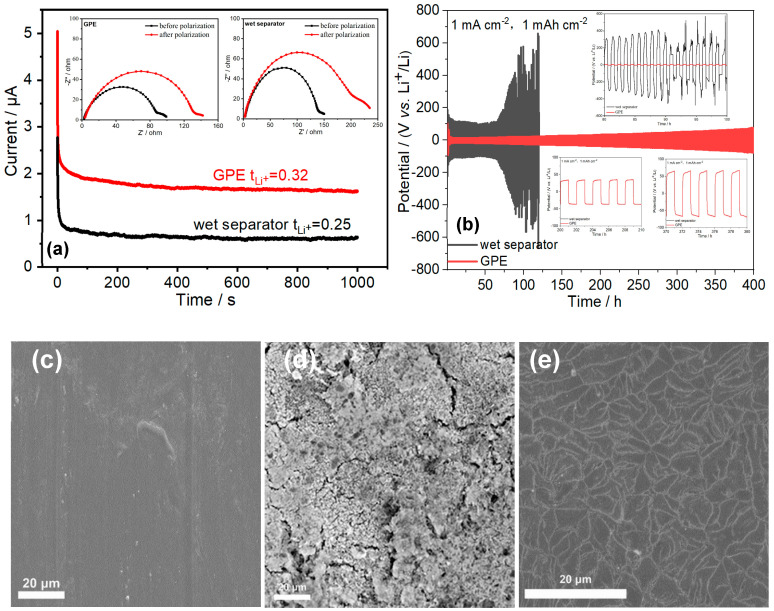
(**a**) Chronoamperometry profiles for PA6/P(VDF-HFP)/PA6-based GPE membrane and wet separator in block cells with Li metal as electrodes at a step potential of 10 mV. The insets are impendence plots of Li/GPE or wet separator/Li before and after polarization. (**b**) The Li^+^ ion deposition/stripping profiles of Li/GPE/Li and Li/wet separator/Li symmetrical cells at a current density of 1 mA cm^−2^; The deposition capacity is 1 mAh cm^−2^. Insets: Detailed plating/stripping voltage profiles at the cycles of 70–110th, 200–210th, and 370–380th. (**c**) SEM micrograph of the surface of Li metal (**c**) before cycling, (**d**) after 120 cycles in Li/wet separator/Li cell at 1mA cm^−2^, and (**e**) after 400 cycles in Li/GPE/Li cell at 1 mA cm^−2^.

The contribution of the lithium cations to the overall ionic conductivity is assessed by the lithium transference number (t_Li+_). The low ratio of charge transported by Li^+^ ions to the overall charge possibly leads to serious concentration polarization, reduced discharge capacity, and other unwanted phenomena [30]. The Bruce–Vincent method is the most widely used method for determining transference numbers in polymer electrolytes [31]. Combined the alternating impedance spectroscopy and the chronoamperometry profile displayed in Figure 4a, the calculated lithium transference numbers of the PA6/P(VDF-HFP)/PA6-based GPE and the wet separator are about 0.32 and 0.25, respectively. The increase of the lithium transference number in GPE can be explained by the formation of a hydrogen bond between PF6^-^ anions and the PA6 or P(VDF-HFP) molecular chains [32]. However, the high absorption to LiPF_6_-based electrolyte makes the increase of the Li^+^ transference number in GPE non-significant.

Li metal has been regarded as the holy grail anode for rechargeable battery systems with high energy density. However, the development of the Li anode has been hampered for decades by dendrite formation during cycling. The nucleation and growth of Li metal dendrites are now considered to be complex multistep reactions [31]. Among the reactions, the nonuniform Li^+^ ion plating/stripping between the electrolyte and Li anode is thought to be the key step of Li dendrite growth [33]. Along with the inhomogeneous electrodeposition process, the nucleation of the Li dendrite will gradually grow until piercing through the separator, then leading to internal short circuits, and thus overheating and fires. For evaluating the stability of the lithium plating/stripping, a galvanostatic cycling test was operated with the Li/GPE or wet separator/Li symmetric cells at a current density of 1 mA cm^−2^ and an areal capacity of 1.0 mAh cm^−2^. The obtained time-dependent voltage profiles of the symmetric cells are shown in Figure 4b. In the case of the Li/GPE/Li cell, the relatively high-voltage polarization appeares in the initial several cycles due to the electrode activation and the formation of an SEI (solid electrolyte interface) layer. Then, the cell exhibits a stable cycle over 400 h with a low polarization potential difference under 70 mV (the insets in Figure 4b), indicating the stable lithium plating/stripping process between the electrolyte and Li metal anode and good electrolyte/electrode interfacial compatibility, which confirms the effective suppression to Li dendrites of PA6/P(VDF-HFP)/PA6-based GPE. The positive result is also proven by the SEM micrograph of the Li electrode surface after 400 cycles displayed in Figure 4e. Except for some irregular stripes deriving from the smooth SEI layer, the Li metal after long galvanostatic cycling is almost the same as the fresh Li metal, owing to the stable electrodeposition on the surface of the Li electrode. As for the Li/wet separator/Li cell, the polarization voltage increases significantly (bigger than 110 mV) compared to that of the Li/GPE/Li cell at the beginning (the insets in Figure 4b). At the 91^st^ cycle, the polarization voltage decreases suddenly, illustrating the occurrence of the short circus in the Li/wet separator/Li because of the continual growth of metallic dendrites [34]. Shown as the dissemble Li/wet separator/Li cell (Figure 4d), the inhomogeneous SEM layer and lots of lithium dendrites rooting from the nonuniform ion plating/stripping are presented clearly. The good ability of dendrite suppression of the electrospun PA6/P(VDF-HFP)/PA6-based GPE can be interpreted by the precise porosity structure made of the interlaced electrospinning nanofibers enabling uniform Li+ ion flux distribution and electrodeposition [35].

**Figure 5:**
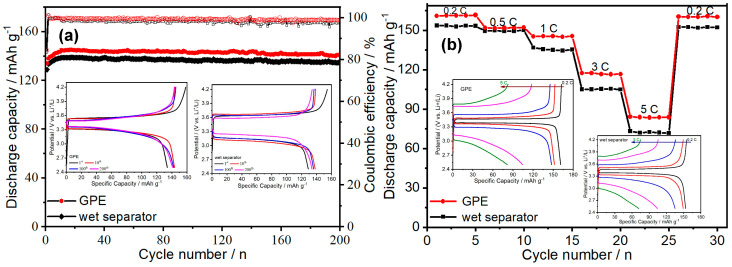
(**a**) The cycle performance of the Li/GPE/LiFePO_4_ cell and the Li/wet separator/LiFePO_4_ cell at 1C. The insets are the charge–discharge curves of the selected cycles of the cells with GPE or wet separator. (**b**) The rate performance of the Li/GPE/LiFePO_4_ cell and the Li/wet separator/LiFePO_4_ cell. The insets are the charge–discharge curves of the selected cycles of the cells with GPE or a wet separator at different current densities.

To explore the feasibility in practical applications, the charge–discharge behaviors of the cells assembled with PA6/P(VDF-HFP)/PA6-based GPE and wet separator using the LiFePO_4_ (LFP) as cathode and Li metal as anode were investigated. The discharge capacities of the cells at 1 C (170 mA g^−1^) are depicted in Figure 5a. With the electrolytes wetting the electrode materials, the discharge capacity of the cells increases little by little and then remains steady. The low Coulombic efficiency in the first cycle proves the formation of SEI. The Li/GPE/LPF cell exhibits good cycle stability and delivers the capacity of 145.5 mAh g^−1^ after 200 cycles, which is higher than that of the cell with a wet separator (134.6 mAh g^−1^). Moreover, the Coulombic efficiency of the Li/GPE/LPF cell is about 98.5% after 200 cycles, indicating the uniform lithium plating/stripping behaviors between the electrolyte and Li metal anode. The charge–discharge curves of cells with GPE or a wet separator in 1st, 10th, 100th, and 200th cycles are displayed in the insets of Figure 5a. It can be seen that the overpotential differences of the selected cycles of the cells with GPE (0.28 mV) are lower than those (0.33 mV) of the cells with wet separators, which testifies to the dynamical stability of the Li/GPE interface during the cycle process.

For further demonstrating the electrochemical performance of the electrospun composite GPE in a real battery system, the rate capabilities of the cells of Li/GPE/LiFePO_4_ and the Li/wet separator/LiFePO_4_ at different currents (0.2 C, 0.5 C, 1 C, 3 C, 5 C, 0.2 C) were evaluated, and the results are shown in Figure 5b. The discharge-specific capacities of the cells with the GPE and wet separator gradually decrease with the increasing of the charge–discharge current, and the reversible discharge capacities of the cells almost recover to the original capacity when the current returns to 0.2 C. However, the Li/GPE/LiFePO_4_ always delivers a higher specific capacity than the Li/wet separator/LiFePO_4_ cell does under the same charge–discharge condition (160.9 mAh g^−1^ versus 152.5 mAh g^−1^ at 0.2 C, 151.8 mAh g^−1^ versus 149.3 mAh g^−1^ at 0.5 C, 145.5 mAh g^−1^ versus 134.6 mAh g^−1^ at 1 C, 116.7 mAh g^−1^ versus 105.3 mAh g^−1^ at 3 C, 83.6 mAh g^−1^ versus 72.1 mAh g^−1^ at 5 C and 160.6 mAh g^−1^ versus 152.4 mAh g^−1^ at 0.2 C), which confirm the excellent rate performance and good suppression to Li dendrites of the PA6/P(VDF-HFP)/PA6-based GPE. Furthermore, the overpotential differences of the charge/discharge voltages at the corresponding current density of Li/GPE/LiFePO_4_ cell are smaller than those of the Li/wet separator/LiFePO_4_ cell (0.09 mV versus 0.11 mV at 0.2 C, 0.17 mV versus 0.19 mV at 0.5 C, 0.28 mV versus 0.33 mV at 1 C, 0.60 mV versus 0.65 mV at 3 C, 0.76 mV versus 0.82 mV at 5 C) because of the excellent interfacial compatibility between the GPE and Li anode.

## 4. Conclusions

In this paper, a PA6/P(VDF-HFP)/PA6 three-layered composite membrane has been fabricated via the layer-by-layer electrospinning technique. The nylon-based composite membrane exhibits high absorption to organic liquid electrolyte (270 wt %) owing to its high porosity (90.35%), good mechanical property (17.11 MPa), and outstanding shut-down behavior from 145 to 230 °C. Moreover, the dimensional shrink of the PA6 porous membrane immersed into liquid electrolyte is cured for the existence of the P(VDF-HFP) middle layer. After swelling by the LiPF_6_-based organic liquid electrolyte, the obtained PA6/P(VDF-HFP)/PA6-based GPE shows high ionic conductivity at room temperature (4.2 mS cm^−1^), a wide electrochemical stable window (4.8 V), and low activation energy for Li^+^ ion conduction (4.68 kJ mol^−1^). Benefiting from the precise porosity structure made of the interlaced electrospinning nanofibers and the superior physicochemical properties of the nylon-based composite GPE, the reversible Li^+^ ion dissolution/deposition behaviors between the GPE and Li anode are successfully realized with the Li/Li symmetrical cells (1.0 mA cm^−2^, 1.0 mAh cm^−2^) proceeding over 400 h at a polarization voltage of no more than 70 mV. Furthermore, the PA6/P(VDF-HFP)/PA6-based GPE in assembled Li/LiFePO_4_ cells displays good electrochemical stability, high discharge capacity, good cycle durability, and high rate capability. This research provides a new strategy to fabricate gel polymer electrolytes via the electrospinning technique for rechargeable Li batteries with good electrochemical performance, high security, and low cost.
